# Effect of Nitrogen on Growth and Optical Properties of Single-Crystal Diamond Synthesized by Chemical Vapor Deposition

**DOI:** 10.3390/ma17061311

**Published:** 2024-03-12

**Authors:** Ying Ren, Wei Lv, Xiaogang Li, Haoyong Dong, Nicolas Wöhrl, Xun Yang, Zhengxin Li, Tao Wang

**Affiliations:** 1 Engineering and Technology Research Center of Diamond Composite Materials of Henan, School of Materials Science and Engineering, Henan University of Technology, Zhengzhou 450001, Chinazhengxin_li@haut.edu.cn (Z.L.); 2 Changan Automobile Global Research and Development Center, Chongqing Changan Automobile Co., Ltd., Chongqing 400054, China; 3 Faculty of Physics and CENIDE, University Duisburg Essen, Carl-Benz-Straße 199, 47057 Duisburg, Germany; nicolas.woehrl@uni-due.de; 4 Henan Key Laboratory of Diamond Optoelectronic Materials and Devices, Key Laboratory of Material Physics, Ministry of Education, School of Physics and Microelectronics, Zhengzhou University, Zhengzhou 450052, China; 5 Avanced Energy Storage Technology Center, Shenzhen Institute of Advanced Technology, Chinese Academy of Sciences, Shenzhen 518055, China

**Keywords:** single-crystal diamonds, high-rate growth, nitrogen, NV defects, microwave plasma CVD

## Abstract

Concurrently achieving high growth rate and high quality in single-crystal diamonds (SCDs) is significantly challenging. The growth rate of SCDs synthesized by microwave plasma chemical vapor deposition (MPCVD) was enhanced by introducing N_2_ into the typical CH_4_-H_2_ gas mixtures. The impact of nitrogen vacancy (NV) center concentration on growth rate, surface morphology, and lattice binding structure was investigated. The SCDs were characterized through Raman spectroscopy, photoluminescence (PL) spectroscopy, and X-ray photoelectron spectroscopy. It was found that the saturation growth rate was increased up to 45 μm/h by incorporating 0.8–1.2% N_2_ into the gas atmosphere, which is 4.5 times higher than the case without nitrogen addition. Nitrogen addition altered the growth mode from step–flow to bidimensional nucleation, leading to clustered steps and a rough surface morphology, followed by macroscopically pyramidal hillock formation. The elevation of nitrogen content results in a simultaneous escalation of internal stress and defects. XPS analysis confirmed chemical bonding between nitrogen and carbon, as well as non-diamond carbon phase formation at 0.8% of nitrogen doping. Furthermore, the emission intensity of NV-related defects from PL spectra changed synchronously with N_2_ concentrations (0–1.5%) during diamond growth, indicating that the formation of NV centers activated the diamond lattice and facilitated nitrogen incorporation into it, thereby accelerating chemical reaction rates for achieving high-growth-rate SCDs.

## 1. Introduction

Recently, there has been a remarkable surge in interest regarding single-crystal diamonds (SCDs) due to their exceptional mechanical, thermal, optical, electrical, and chemical properties [1,2,3]. However, high-pressure- and high-temperature-produced (HTHP) SCDs are not suitable for applications in the domains of optics and electronics due to limitations in size, purity, and cost. The microwave plasma chemical vapor deposition (MPCVD) method is considered to be one of the most promising technologies for producing large-surface-area SCDs with high purity at a low cost. Nevertheless, one significant challenge that needs to be addressed is accelerating the growth rate before achieving high-quality diamond films.

Previous studies have demonstrated that growth rates exceeding 10 μm/h can be achieved by employing high-power-density plasma with a high concentration of methane [4,5]. Yan et al. [6] from the Carnegie Institution have shown that rapid growth rates ranging from 50 to 150 µm/h can be attained using MPCVD by incorporating a small quantity of nitrogen. Chayahara et al. [7] from the Institute of Industrial Technology of Japan (AIST) achieved fast growth rates for SCDs up to 30–120 µm/h with a minor nitrogen addition through the utilization of a specially designed substrate holder. These reports collectively confirm that increasing methane concentration, plasma power density, and adding small amounts of N_2_ can significantly enhance diamond growth rate. However, it should be noted that nitrogen as an impurity atom increases defect content and leads to yellow discoloration in diamonds [8,9,10], which adversely affects their various physical properties such as their optical properties and thermal conductivity [11,12].

Furthermore, the presence of nitrogen results in a significant modification of the surface composition of adsorbed species, leading to the formation of a coarse surface morphology [13]. However, this morphological alteration does not facilitate further growth without time-consuming mechanical polishing processes. Otherwise, it would readily result in the formation of twins and/or polycrystalline structures, thereby compromising the quality of single-crystal diamonds (SCDs). Therefore, it is crucial to investigate how the accelerated growth rates and changes in specific properties induced by nitrogen addition balance each other out.

Currently, there is an ongoing debate regarding the cause of the significant increase in diamond growth rate solely due to nitrogen addition. Some researchers have proposed that this increased growth rate can be attributed to the catalytic effect of nitrogen [14]. Specifically, a nitrogen-containing species (simplified as CN radicals) initially competes with hydrocarbon species for free radical sites and subsequently catalyzes the incorporation of hydrocarbon growth species into the diamond lattice within its vicinity. Furthermore, nitrogen addition leads to an increase in carbon supersaturation, thereby enhancing reaction rates on the growth surface and consequently promoting diamond deposition rates [15]. Recent studies on doped nitrogen have confirmed that predominantly substitutional nitrogen (NS) defects are present in diamonds, accompanied by a lower proportion of NV defects [16,17]. Numerous theoretical investigations have primarily focused on substitutional nitrogen’s impact, revealing that unpaired electrons from subsurface NS diffuse near the growing surface and weaken surrounding bonds, thus increasing surface reactivity [18]. The effects of nitrogen on the structure and properties of carbon-based materials were calculated using first principles based on density functional theory (DFT), which have successfully guided the experiments [19,20]. Additionally, excess energy carried by these subsurface NS electrons reduces overall energy requirements for hydrogen abstraction and methyl molecule adsorption, resulting in higher concentrations of active growth sites on the surface [21,22]. These factors contribute to accelerated growth when incorporating nitrogen. However, limited research has been conducted to thoroughly explore how varying concentrations of NV defects introduced through different levels of nitrogen addition affect single-crystal diamond development. 

This paper presents the synthesis of single-crystal diamonds (SCDs) through the addition of varying concentrations of N_2_ to the conventional CH_4_-H_2_ gas mixtures in a microwave plasma chemical vapor deposition (MPCVD) system. Our objective is to investigate the impact of nitrogen doping on the high-rate growth of SCDs by examining changes in NV-related defect concentrations. Additionally, we elucidate alterations in surface morphology, microscopic bond structure, and optical properties of CVD SCDs as a function of N_2_ flow rate.

## 2. Materials and Methods

The growth of SCDs was conducted using a 5 kW, 2.45 GHz microwave plasma chemical vapor deposition (MPCVD) system (Zhengzhou Tianhong Automation Technology Co., Ltd., Zhengzhou, China). The experimental conditions are summarized in Table 1. High-temperature, high-pressure Ib (100) SCDs (Henan Huanghe Whirlwind Co., Ltd., Changge, China), commercially synthesized and measuring 3.8 × 3.8 × 1 mm^3^, were utilized as seed crystals after undergoing ultrasonic cleaning with ethanol and acetone for a duration of 15 min each. This was followed by plasma etching for surface defect elimination in the MPCVD reactor at a pressure of 12 KPa with H_2_ flow rates of 300 sccm (Standard Cubic Centimeter per Minute) for a period of thirty minutes prior to diamond growth initiation. During the four-hour growth process, fixed H_2_ and CH_4_ flow rates were maintained at values of 300 sccm and 24 sccm, respectively; however, N_2_ flow rates varied between samples at values ranging from zero to increments such as 0.6 sccm, 1.5 sccm, 2.4 sccm, 3.6 sccm, or 4.5 sccm to investigate the impact on SCD growth behavior caused by nitrogen addition. The six samples were named S0, S1, S2, S3, S4, and S5, respectively, in accordance with their corresponding N_2_ flow rates.

The surface morphologies of the grown diamonds were characterized using a Raman confocal Olympus microscope (HORIBA Jobin Yvon, Longjumeau, France), and the pinhole three-dimensional confocal method was adopted with a horizontal spatial resolution of 350 nm (HORIBA Jobin Yvon, Longjumeau, France). The crystalline quality and optical properties were assessed through room-temperature Raman spectroscopy and photoluminescence (PL) spectroscopy, utilizing a micro-Raman HORIBA Jobin Yvon (HORIBA Jobin Yvon, Longjumeau, France) LabRAM HR 800 visible spectrometer equipped with a Peltier-cooled CCD detector with a 532 nm Nd and a YAG laser of about 50 mW. The spectral resolution was less than or equal to 0.4 cm^−1^. The spectral range was 535~1050 nm for PL spectroscopy. X-ray photoelectron spectroscopy was employed to analyze the microscopic bond structure in the synthesized SCDs using Al Ka (1486.6 eV) radiation in Thermo Scientific ESCALAB 250Xi (ThermoFischer, Waltham, MA, USA), under a base pressure of 8 × 10^−8^ Pa. The full spectrum of the test-passing energy was 50 eV, while the narrow spectrum was limited to 20 eV with a step size of 0.05 eV. The sample’s charge was adjusted based on the C1s (285 eV) binding energy, as the energy standard.

## 3. Results and Discussion

**Figure 1:**
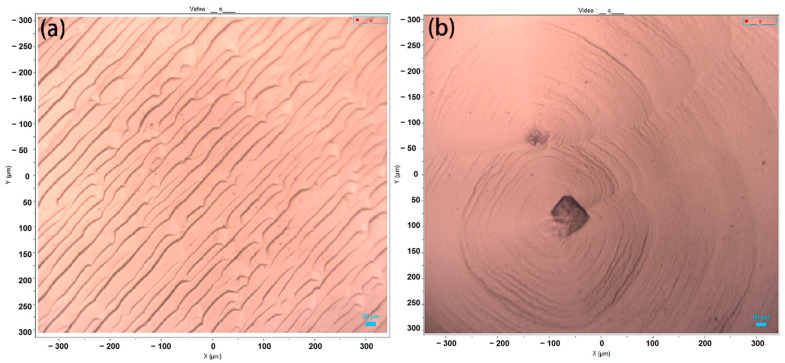
Raman confocal microscope images of the CVD SCDs deposited without and with N_2_ addition: (**a**) S0; (**b**) S2.

The surface morphology of homoepitaxial single-crystal diamonds is presented in Figure 1, before and after the introduction of N_2_. It can be observed that, without nitrogen addition (S0, Figure 1a), the diamond exhibits a typical step–flow growth mode [23]. However, upon adding N_2_ to the source gas, pyramidal hillocks with round corners similar to S2 (Figure 1b) are formed, accompanied by the generation of diamond particles. The inclusion of nitrogen leads to an increased step density and roughness on the surface of sample due to an increase in supersaturation of developing species on the surface [15]. This hinders step propagation as it reduces the reactivity of the surface nitrogen atoms [24].

Hence, when the step width is comparable to the diffusion distance of radical species, the growth species collide, stack, and nucleate in the middle of steps instead of diffusing along step edges. This phenomenon leads to two-dimensional nucleation which results in higher adsorption of species and subsequently creates shorter steps around the edge of the cores. Consequently, a long-period locally dominant growth forms hillocks that are topped with diamond particles. These developed hillocks serve as sources for steps and contribute to the observed step-bunching phenomenon. Therefore, it can be inferred that the growth mode of diamond transitions from step–flow to bi-dimensional nucleation. This finding aligns with Achard et al.’s report [25].

**Figure 2:**
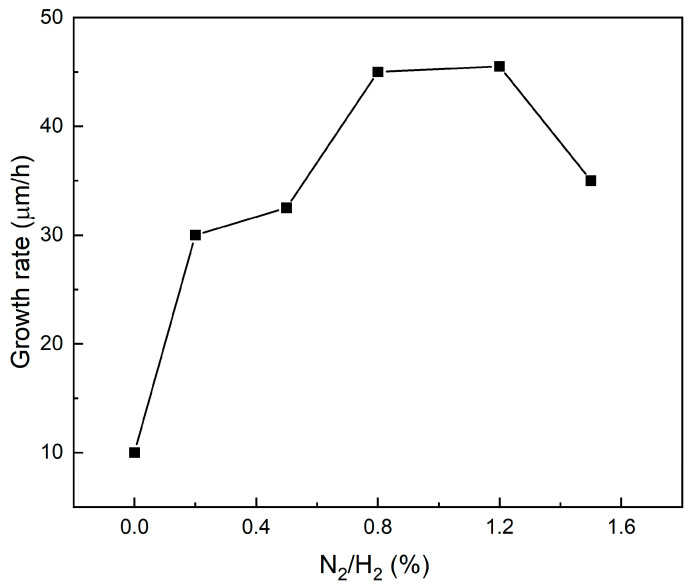
Growth rates of the samples versus levels of nitrogen doping.

The growth rates of the samples as a function of nitrogen concentration are illustrated in Figure 2. Under the typical deposition conditions without N_2_ addition, the growth rate is approximately 10 μm/h (sample S0). Upon introducing 0.2% of N_2_, there is a significant increase in the growth rate from 10 μm/h to 30 μm/h (Sample S1). Subsequently, with the nitrogen content gradually increasing to 0.8% and 1.2%, the growth rates progressively rise and stabilize at 45 μm/h for S3 and 45.5 μm/h for S4, representing an enhancement by a factor of 4.5 compared to S0 without nitrogen. However, at a higher N_2_ concentration of 1.5%, there is an inverse reduction observed in the growth rate for S5. Similar trends have been previously reported for both polycrystalline [13] and single-crystal diamond [7,26] growth.

According to Frauenheim’s model [27], it is predicted that the substitutional subsurface nitrogen (N_S_) plays a vital role in activating the diamond lattice and expediting the incorporation of growth units along dimer columns on (100) faces, thus supplying additional electron energy. The interaction between the dominant substitutional nitrogen (N_S_) defects and surficial carbon atoms has been convincingly confirmed [16,17,28]. This discovery aligns with the proposed model and elucidates why even a minute quantity of added nitrogen significantly boosts growth rate. Similarly, when a sufficient number of electrons are supplied to saturate surface dangling bonds, it can be inferred that growth rate reaches a plateau. The diminished deposition rate of diamond at higher levels of nitrogen doping (1.5% for S5) can be ascribed to excessive unpaired electrons provided by substitutional nitrogen, which significantly dehydrogenates the growing surface and results in an excess of adjacent unsaturated bonds; meanwhile, insufficient bonding with the hydrocarbon growth precursor occurs. 

Therefore, the diamond surface’s suspended bonds collapse and undergo a transformation into graphite bonds, which may deteriorate the crystalline quality of the diamond. Furthermore, due to the high bond energy of carbon and nitrogen, desorbing nitrogen atoms from CN bonds on the surface to create new growth sites becomes even more challenging compared to CH bonds [29]. These factors collectively impede further combination with diamond growth precursor, explaining why a higher N_2_ gas mixture ratio does not always enhance high-rate growth and why there is a saturation in the deposited rate of SCDs. However, nitrogen in its existing forms encompasses not only NS but also NV centers, albeit in small proportions. The influence of NV centers on deposition and growth processes as well as on the crystalline quality of diamonds is equally indispensable.

**Figure 3:**
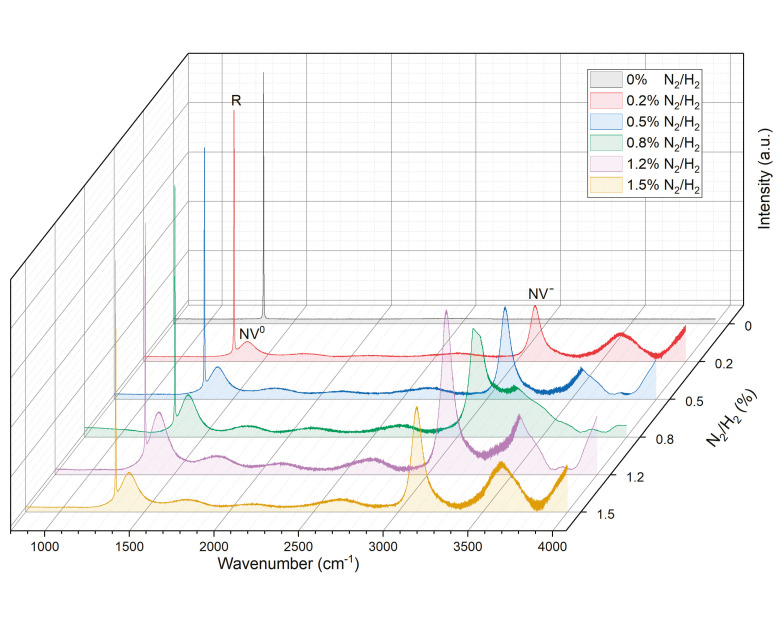
Raman spectra taken from the CVD SCDs deposited with different levels of nitrogen doping.

The Raman spectra were examined at room temperature to investigate the quality of synthesized diamond with varying nitrogen concentrations in the gas mixture. As illustrated in Figure 3, the prominent and well-defined peak at 1332 cm^−1^ corresponds to the intrinsic zone center phonon band of a diamond, which is observed in all spectra. Upon introduction of N_2_, samples S1–S5 exhibit new peaks at 1410 cm^−1^ and 3110 cm^−1^. Conversion of wavenumber into wavelength reveals that these peaks correspond to 575 nm and 637 nm, respectively, representing (N-V)0 and (N-V) centers (where NV denotes a nitrogen atom occupying a carbon site adjacent to a vacancy in the diamond carbon lattice) [30]. The background within the range of 3500~3700 cm^−1^ can be attributed to sidebands of zero phonon line (ZPL), associated with (N-V)^−^ centers [31]. The presence of NV-related bands in the spectra confirms nitrogen incorporation into deposited SCDs for samples S1–S5. Normalization of the Raman spectrum curves against the first-order Raman peak (R) at 1322 cm^−1^ demonstrates synchronous changes between emission intensity of NV centers and deposition rate, as depicted in Figure 2. This suggests a significant correlation between the concentration of NV centers and the rate of SCD expansion, and will be further discussed in the PL spectral results.

**Figure 4:**
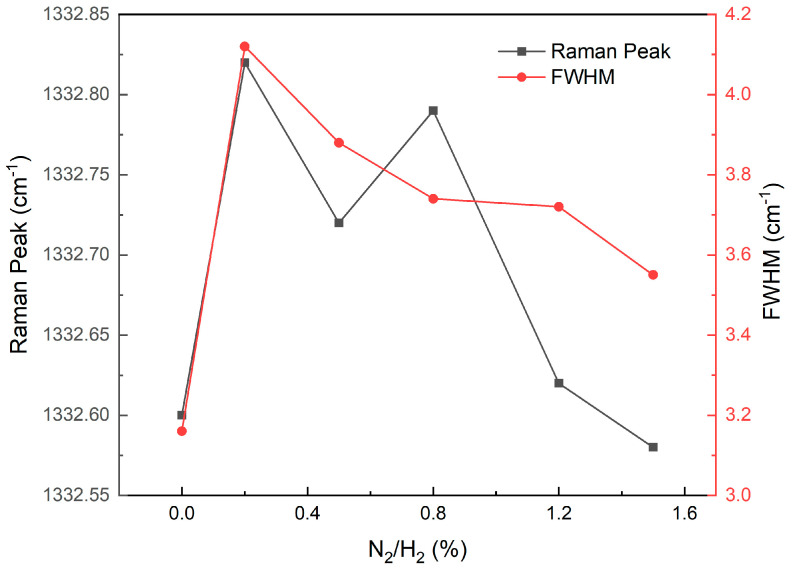
Raman characteristic peak position and FWHM taken from diamonds deposited with different levels of nitrogen doping.

The Raman peak position of the diamond at around 1332 cm^−1^ and its full-width-at-half-maximum (FWHM) provide insights into the crystalline characteristics and presence of strain in the films. As depicted in Figure 4, the FWHM initially increases and then decreases with increasing levels of nitrogen doping from 0% to 1.5%, exhibiting a similar trend for the peak position except for S3. This suggests that compressive stress is induced due to lattice expansion caused by N-related defects [32], which subsequently relaxes as nitrogen concentration and film thickness increase. It should be noted that complete elimination of the improved ionic nature of C–N bonds compared to C–C bonds within the diamond lattice may not be feasible [33]. Furthermore, considering the contribution of the (N-V)^0^ peak near the characteristic diamond peak to peak broadening, it can be concluded that there is no significant degradation in sample crystallinity upon the addition of N_2_.

**Figure 5:**
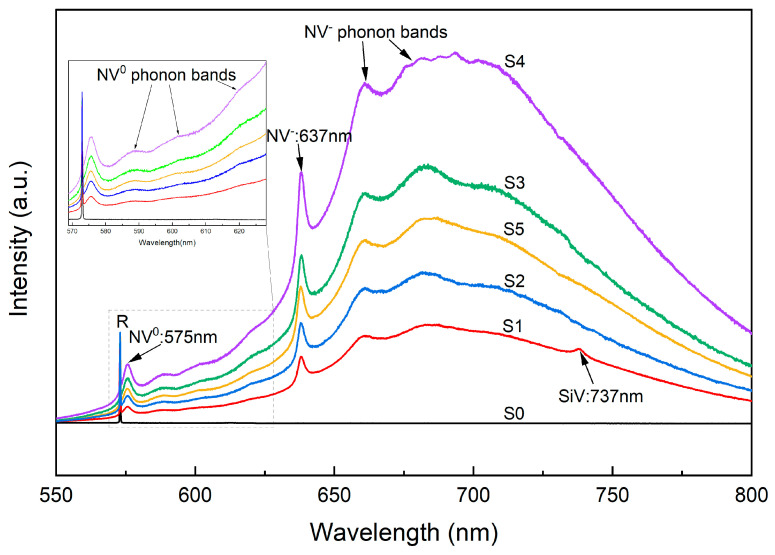
PL spectral of synthesized SCDs with different levels of nitrogen doping. Inset: magnification of part of PL spectra in the 570–625 nm range (excitation, 300 K). The intensities were normalized to the diamond first-order Raman peak.

To further investigate the efficacy of NV center generation in the synthesized SCD samples and its concentration in enhancing growth rate, PL spectral evolution examination was conducted as shown in Figure 5. The intensities of the curves were normalized to the diamond first-order Raman peak (R) at 572 nm. The typical zero phonon lines (ZPL) observed at 575 and 637 nm were exclusively present in samples S1–S5 with N_2_ addition from 0.2% to 1.5%; this is consistent with the Raman spectra depicted in Figure 3, which corresponded to the neutral (N-V)^0^ and negatively charged states (N-V)^−^ of nitrogen vacancy centers. The presence of silicon vacancy (Si-V) centers at 737 nm [34] can be attributed to quartz windows etching exposed to high-heat plasma inside the CVD chamber [34,35]. It was observed that, for sample S1 with a nitrogen concentration of 0.2%, there was an insignificant Si-V center signal compared to sample S0 without any nitrogen addition, while no Si-V center signal could be detected for samples S2–S5. With higher levels of nitrogen doping, nitrogen tends to exist in clusters rather than isolated substitutional form, thereby deteriorating diamond quality and potentially altering bonding and structure formation along with other defects such as N_2_ clusters coexisting with Si-V centers, ultimately leading to the quenching of Si-V luminescence [36].

Hence, the absence of a Si-V center for S2–S5 can be explained. Furthermore, two other intense broad bands in the region of 580–620 nm and 650–725 nm are assigned to ZPL peaks related to (N-V)^0^ and (N-V)^−^ defects, respectively [37]. The PL spectra intensity of NV-related peaks and bands gradually increases with N_2_ addition, reaching a maximum at 1.2% N_2_ concentration for S4. However, as the N_2_ concentration increases to 1.5% for S5, the intensity decreases significantly, indicating reduced nitrogen atom trapping by vacancies at excessive nitrogen concentrations. 

Notably, the emission intensities of NV centers exhibit similar trends to the growth rate shown in Figure 3. The NV-related emissions monotonously increase with increasing NV concentration [16,28]. Therefore, it can be inferred that the increased growth rate corresponds to an augmentation in NV concentration through nitrogen doping. Previous studies have demonstrated that vacancy-assisted diffusion is energetically favored over direct interstitial or exchange mechanisms in diamonds [36,37,38,39]; these results suggest a decrease in the charged vacancy diffusion activation barrier, which enhances self-diffusion in doped diamond crystals. Thus, we conclude that an increasing number of nitrogen atoms have been incorporated into the diamond crystal lattice through vacancy-assisted diffusion for S1–S4 samples; among these samples, the (N-V)^−^ defect form predominates during the diamond growth process. This observation is consistent with the significant enhancement achieved by nitrogen doping, as shown in Figure 2.

At a higher level of nitrogen doping of 1.5% for S5, the intensity of NV defects correspondingly decreases in accordance with the diminished rate. We propose three possible reasons to explain the phenomenon. Firstly, it has been observed that the typical doping efficiency of N in diamond is lower at a magnitude of 10^−4^ [16,40]. Additionally, part of the doped N in diamond crystallite may exist as N aggregates rather than substitutional N or NV centers at higher levels of nitrogen doping [36,41]. Secondly, it is known that the concentration of NV defects is limited by vacancies [16,40], implying that, during diamond growth through n- or p-type doping, there will be a decrease in the production of vacancies or other point defects due to the Fermi level shifting towards conduction or valence bands through doping. This results in negatively and positively charging vacancies, respectively [42]. Consequently, the lower concentration of carbon vacancy production due to nitrogen doping restricts further augmentation of NV centers. Finally, our experiments were conducted with a substrate temperature around 950 °C; however, slightly higher substrate temperatures (over 50 °C) were observed for S5 under similar microwave power and pressure conditions as the other samples. It has been demonstrated that an increase in substrate temperature leads to a decline in NV formation efficiency [43].

All these analyses suggest that reduced NV centers indicate a possibility where nitrogen incorporation into SCDs crystal lattice decreases. As a result, saturation occurs when nitrogen doping reaches its limit through vacancies diffusion within the diamond lattice structure leading to decreased content of NV centers due to excessive nitrogen doping. The influence exerted by nitrogen on reaction facilitation weakens with fewer new growth sites and lower reaction rates on the growth surface, which explains why there is synchronous change between growth rate and concentration of NV centers for SCDs.

**Figure 6:**
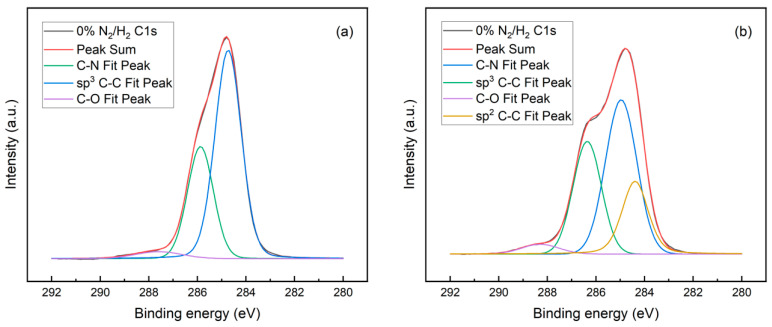
XPS C1s spectra of synthesized SCDs with (**a**) 0% and (**b**) 0.8% N_2_ addition.

To further quantify the microscopic changes in bond structure of SCDs following the introduction of nitrogen gas concentrations, XPS measurements were conducted, as shown in Figure 6 for samples S0 and S3. The observed alterations in the symmetry and width of C1’s peaks indicate variations in both the microstructure and the bond structure of carbon upon nitrogen flow addition. Specifically, a characteristic peak at approximately 284.4 eV corresponds to sp2 C–C bonds, while another peak at around 285 eV corresponds to sp3 C–C bonds within the XPS C1s spectrum. With 0.8% of nitrogen doping, the emergence of non-diamond carbon with sp2 C–C bonding is consistent with the obtained Raman results.

Additionally, peaks at approximately 286 eV and 287.8 eV can be attributed to C–N and C–O bonds, respectively; their relative intensities are related to their respective areas under the XPS spectrum [44], indicating that both types of bonds expand with nitrogen addition. It should be noted that, even without nitrogen addition (S0 sample), traces of C–N bonding were still observed, possibly originating from the impurities present in gas sources such as methane (99.999%) or due to low base leakage rates during synthesis processes. Similar observations were made regarding the presence of a C–O bond within this sample set as well. The chemical bonds formed by small amounts of nitrogen or oxygen with carbon can be calculated using first principles based on density functional theory (DFT) [20].

The XPS N1s spectrum exhibited only one peak at approximately 400 eV for both samples containing either 0% or 0.8% N_2_ additions; this peak can be attributed to the presence of a C–N bond, as shown in Figure S1. These findings suggest that nitrogen indeed forms a binding interaction with carbon through a process involving formation of a C–N bond, leading to the introduction of non-diamond phases characterized by sp2 C–C bonding—all these findings are consistent with our Raman spectroscopy results.

## 4. Conclusions

The high-speed growth rate of single-crystal diamonds was achieved by introducing a small amount of nitrogen into the reactant. Nitrogen addition increased the growth rate of homoepitaxial single-crystal diamonds by a factor of 4.5, ranging from 10 to 45 μm/h. The introduction of nitrogen resulted in the observation of a typical step-bunching morphology, which enhanced both step density and surface roughness, indicating a shift in diamond growth mode from step–flow to bi-dimensional nucleation. The shift in the position of the diamond Raman peak diamond indicates the introduction of compressive stress into the diamond lattice through nitrogen addition, and this compressive stress decreases with increasing nitrogen concentration. The XPS analysis confirmed that nitrogen exists as C–N bonds and the diamond lattice consisted of both sp^2^ and sp^3^ C–C bonds due to N doping. Despite relatively minor variations (ranging from 3.1 to 4.1 cm^−1^) around the 1332 cm^−1^ Raman peak, there was no significant deterioration in synthesized diamond quality. The intensity of NV-related defects emission changed synchronously with alternations in growth rate, suggesting that NV-related centers facilitated diamond crystallite stimulation and facilitated the easier integration of nitrogen into the lattice, thereby contributing to improved growth rate and typical step-bunching surface morphology.

## Figures and Tables

**Table 1:** The growth conditions of single-crystal diamonds with N_2_ addition at different concentrations.

Samples	Level of Nitrogen Doping (N_2_/H_2_)/%	CH_4_ Flow Rates/sccm	Time/h	Temperature/°C	Power/kW	Pressure/kPa
S0	0	24	4	~950	~3.25	~13
S1	0.2					
S2	0.5					
S3	0.8					
S4	1.2					
S5	1.5					

## Data Availability

Data are contained within the article.

## Supplementary Materials

The following supporting information can be downloaded at: https://www.mdpi.com/article/10.3390/ma17061311/s1, Figure S1: XPS N1s spectra of synthesized SCDs with (**a**) 0% and (**b**) 0.8% N_2_ addition.
