# Supplementary figures and images for: Effect of Nitrogen on Growth and Optical Properties of Single-Crystal Diamond Synthesized by Chemical Vapor Deposition
Source: Materials (Basel). 2024 Mar 12;17(6):1311. doi: 10.3390/ma17061311 (PMC10972215; doi:10.3390/ma17061311)

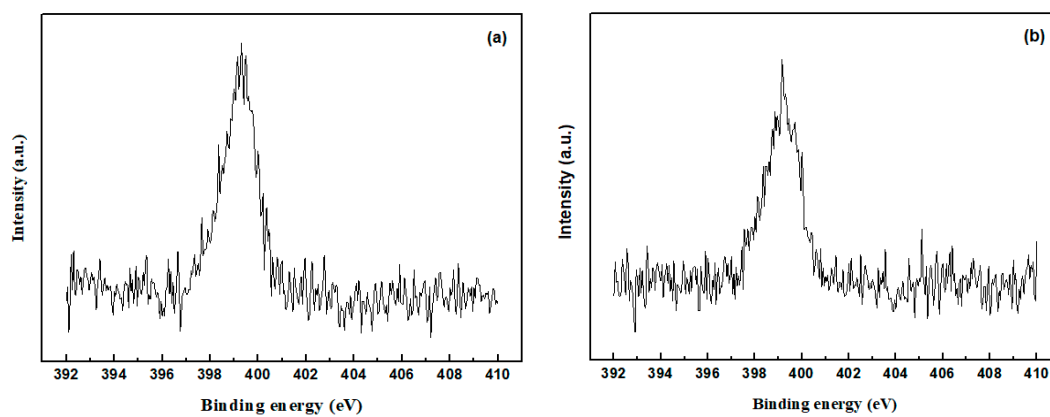

**Figure S1.** XPS N1s spectra of synthesized SCDs with (a) 0% and (b) 0.8% N<sub>2</sub> addition.

Supplement: Supplementary file 1 [file materials-17-01311-s001.zip › materials-2906372-supplementary.pdf]
